# hsa-miR-20b-5p and hsa-miR-363-3p Affect Expression of *PTEN* and *BIM* Tumor Suppressor Genes and Modulate Survival of T-ALL Cells In Vitro

**DOI:** 10.3390/cells9051137

**Published:** 2020-05-05

**Authors:** Monika Drobna, Bronisława Szarzyńska, Roman Jaksik, Łukasz Sędek, Anna Kuchmiy, Tom Taghon, Pieter Van Vlierberghe, Tomasz Szczepański, Michał Witt, Małgorzata Dawidowska

**Affiliations:** 1 Institute of Human Genetics Polish Academy of Sciences, 60-479 Poznań, Poland; monika.drobna@igcz.poznan.pl (M.D.); bronislawa.szarzynska@pbkm.pl (B.S.); michal.witt@igcz.poznan.pl (M.W.); 2 Department of Systems Biology and Engineering, Silesian University of Technology, 44-100 Gliwice, Poland; roman.jaksik@polsl.pl; 3 Department of Microbiology and Immunology, Zabrze, Medical University of Silesia in Katowice, 41-808 Zabrze, Poland; lsedek@sum.edu.pl; 4 Department of Diagnostic Sciences, Ghent University, 9000 Ghent, Belgium; anna.kuchmiy@ugent.be (A.K.); tom.taghon@ugent.be (T.T.); 5 Cancer Research Institute Ghent (CRIG), 9000 Ghent, Belgium; pieter.vanvlierberghe@ugent.be; 6 Department of Biomolecular Medicine, Ghent University, 9000 Ghent, Belgium; 7 Department of Pediatric Hematology and Oncology, Zabrze, Medical University of Silesia in Katowice, 41-800 Zabrze, Poland; szczep57@poczta.onet.pl

**Keywords:** oncogenic miRNAs, acute lymphoblastic leukemia, silencing tumor suppressor genes, miRNA cluster, noncoding RNAs in cancer

## Abstract

T-cell acute lymphoblastic leukemia (T-ALL) is an aggressive malignancy arising from T lymphocyte precursors. We have previously shown by miRNA-seq, that miRNAs from the mir-106a-363 cluster are overexpressed in pediatric T-ALL. In silico analysis indicated their potential involvement in the regulation of apoptosis. Here, we aimed to test the hypothesis on the pro-tumorigenic roles of these miRNAs in T-ALL cells in vitro. We demonstrate, for the first time, that hsa-miR-20b-5p and hsa-miR-363-3p from the mir-106a-363 cluster, when upregulated in T-ALL cells in vitro, protect leukemic cells from apoptosis, enhance proliferation, and contribute to growth advantage. We show, using dual luciferase reporter assays, Ago2-RNA immunoprecipitation, RT-qPCR, and Western blots, that the oncogenic effects of these upregulated miRNAs might, at least in part, be mediated by the downregulation of two important tumor suppressor genes, *PTEN* and *BIM*, targeted by both miRNAs. Additionally, we demonstrate the cooperative effects of these two miRNAs by simultaneous inhibition of both miRNAs as compared to the inhibition of single miRNAs. We postulate that hsa-miR-20b-5p and hsa-miR-363-3p from the mir-106a-363 cluster might serve as oncomiRs in T-ALL, by contributing to post-transcriptional repression of key tumor suppressors, *PTEN* and *BIM*.

## 1. Introduction

T-cell acute lymphoblastic leukemia (T-ALL) is an aggressive, highly heterogeneous type of lymphoid malignancy, accounting for approximately 15% of pediatric and 25% of adult acute lymphoblastic leukemia (ALL) cases. T-ALL arises from T-cell precursors (thymocytes) during their maturation in the thymus upon accumulation of numerous genetic lesions and cooperating aberrations affecting epigenetic and post-transcriptional regulation of gene expression [1,2,3,4,5,6]. Thus, T-ALL is characterized by a multiplicity of chromosomal rearrangements, copy number alterations, and sequence mutations, leading to activation of oncogenes and inactivation of tumor suppressor genes, which ultimately result in altered gene expression profiles, as a consequence of triggering of oncogenic pathways and transcriptional programs [5,6]. These collectively lead to the dysfunction of key processes involved in the maturation of thymocytes and cause a block of differentiation, the impairment of apoptosis and cell cycle, as well as excessive proliferation. Among the genetic aberrations observed in T-ALL, there are lesions occurring commonly in many other cancers (e.g., inactivating mutations and deletions of *PTEN* and *TP53* genes, activating gene mutations of the *JAK-STAT* pathway) as well as T-ALL-specific lesions, mainly affecting transcription factors crucial for normal differentiation of T-cell precursors (e.g., *BCL11B*, *TAL1*, *TAL2*, *LYL1*).

With such a complex molecular background, T-ALL presents as a highly heterogeneous malignancy. This heterogeneity is observed at the immunophenotypic, genetic and potentially also at the epigenetic level. Better understanding of the biology of this disease, the identification of novel components of oncogenic pathways and potentially druggable targets are needed for further improvements in T-ALL treatment strategies. The recent effort is to exploit the possibilities offered by next generation sequencing (NGS) technologies, including those extending beyond the coding part of the genome, to further unravel the complexity of the (epi)genomic and transcriptomic background of T-ALL [7,8,9,10,11,12] and to understand the functional consequences of these aberrations.

miRNAs constitute a class of non-coding RNAs, extensively studied for their contribution to tumorigenesis by their involvement in the regulation of key cellular functions such as cell proliferation, apoptosis, migration, and many others. miRNAs are involved in the negative regulation of gene expression at post-transcriptional level in health and disease. When aberrantly expressed, miRNAs may act as oncogenes or as suppressors of neoplastic transformation, by silencing the expression of tumor suppressor mRNAs or by insufficient silencing of oncogenic mRNAs, respectively [2]. The biological role of miRNAs lies in their involvement in intricate networks of regulatory interactions: miRNA-mRNA interactions, but also co-operative actions of many miRNAs and interactions with other non-coding RNAs [2,13,14]. 

miRNA-mediated regulation of gene expression is a complex phenomenon. Single miRNA may target multiple mRNAs, and single mRNA may contain MRE (miRNA responsive element) sequences for many miRNAs. miRNAs often act as ‘functional groups’ co-regulating the same cellular processes, e.g., clusters of miRNAs transcribed as polycistronic primary transcript or structurally unrelated but co-expressed and functionally related miRNAs. Such clustered and co-expressed miRNAs collectively contribute to phenotypic effects. Thus, the oncogenic or tumor suppressor potential of particular miRNA is rarely the effect of targeting single mRNA, but is more the ‘net-effect’ of the regulation of multiple targets by several co-operating miRNAs [2]. This also implies that the biological effects of a single miRNA on the expression of a single target gene are usually mild. Thus, the “fine-tuning” regulatory effects of miRNAs over mRNA transcriptome should be studied in a broad context. Next generation sequencing enabling the global investigation of miRNA expression, followed by extensive in silico analysis of related mRNAs and biological processes, is currently an optimal option to select candidate miRNAs for functional analyses. 

The miRNA transcriptome in pediatric T-ALL has been recently comprehensively characterized by NGS [11] also by our group [12]. We identified a set of differentially expressed miRNAs in primary pediatric T-ALL cases as compared to normal mature T-cells. Among miRNAs overexpressed in T-ALL, we found four miRNAs (hsa-miR-20b-5p, miR-20b-3p, miR-18b-5p and hsa-miR-363-3p) that belong to the mir-106a-363 cluster. This miRNA cluster is a paralog of mir-17-92, a prototypic oncogenic cluster of an eminent role in many types of cancer, including T-ALL [15]. Interestingly, miRNAs from mir-17-92 cluster were not overexpressed in our T-ALL cohort. The role of miRNAs from the mir-106a-363 cluster has not been extensively studied in the context of T-ALL. 

In the present study, we aimed to test the hypothesis on the oncogenic roles of hsa-miR-20b-5p and hsa-miR-363-3p, both from mir-106a-363 cluster, in T-ALL cells in vitro. 

We demonstrate that hsa-miR-20b-5p and hsa-miR-363-3p affect the expression of two important tumor suppressor genes, *PTEN* and *BIM*. We also show that mimicry and inhibition of these miRNAs have impact on the survival of T-ALL cells in vitro, by affecting spontaneous apoptosis and proliferation. Thus, we postulate that hsa-miR-20b-5p and hsa-miR-363-3p, might act as oncomiRs when overexpressed in T-ALL and we indicate *PTEN* and *BIM* as potential important mediators of these effects.

## 2. Materials and Methods

### 2.1. miRNA Selection and Target Prediction

The selection of miRNAs analyzed in the present study (hsa-miR-20b-5p, hsa-miR-363-3p) was based on their overexpression in primary T-ALL samples (34 pediatric T-ALL cases and an independent cohort of 32 pediatric T-ALL cases in the validation cohort) as compared to normal mature T-cells, CD34+, and CD4+CD8+ normal thymocytes, as described previously [12,16]. 

Target genes analyzed in the present study were selected based on target prediction and pathway enrichment analysis, performed for miRNAs differentially expressed between T-ALL samples and controls in the miRNA-seq study [12]. Briefly, 8 target prediction algorithms, 3 databases of validated miRNA-mRNA interactions and 3 databases of miRNA-mRNA interactions related to diseases and drug response were used. Genes predicted as targets for differentially expressed miRNAs by more than 5 algorithms were then analyzed for enrichment in Gene Ontology (GO) and Kyoto Encyclopedia of Genes and Genomes (KEGG) terms and pathways. For the details of the target prediction and overrepresentation analysis, refer to our previous work [12].

### 2.2. Primary T-ALL Samples and Control Samples

T-ALL cells were isolated by immunomagnetic selection from bone marrow mononuclear cells obtained at primary diagnosis, as previously described [12]. Bone marrow samples were collected from T-ALL patients and from 5 healthy unrelated bone marrow donors aged <18 years with the informed consent of the patients/legal guards, in accordance with Declaration of Helsinki. Samples were collected at the centers of Polish Pediatric Leukemia and Lymphoma Study Group. The study was approved by the Ethics Committee of the Medical University of Silesia (KNW/0022/KB1/145/I/11/12 and KNW/0022/KB1/153/I/16/17).

**Figure 1:**
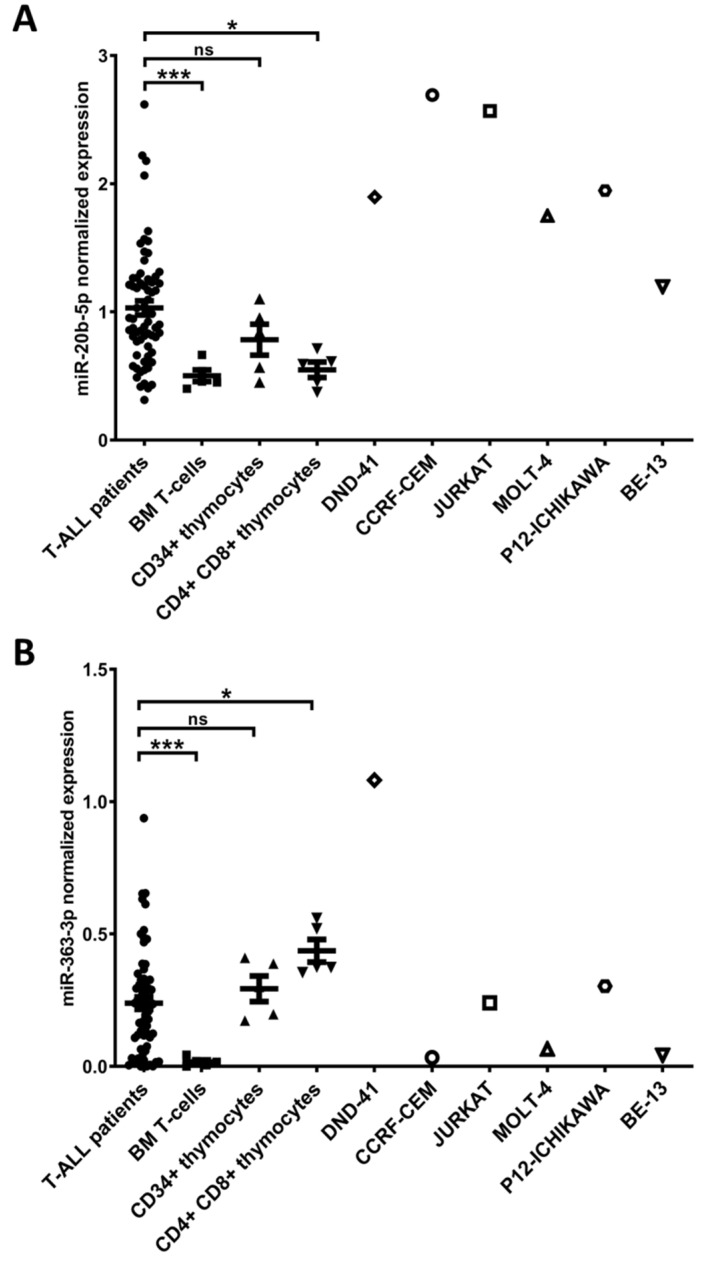
Expression of hsa-miR-20b-5p (**A**) and hsa-miR-363-3p (**B**) evaluated by RT-qPCR in T-cell acute lymphoblastic leukemia (T-ALL) patients, normal T-cells from bone marrow (BM T-cells), CD34+ thymocytes, CD4+ CD8+ thymocytes and T-ALL cell lines. *** *p* < 0.001; * *p* < 0.05; ns—not significant.

Thymocyte samples, obtained as previously described [11,17] were used as controls. RNA isolated from thymocytes (3 CD34+ and 3 CD4+CD8+) was used in RT-qPCR expression analysis of the studied miRNAs in T-ALL primary samples and 6 T-ALL cell lines, to extend the previous validation [12,16] (Figure 1). Human thymus samples were used following the guidelines of, and were approved by, the Ethical Committee of the Ghent University Hospital (Belgium).

### 2.3. Cell Lines

The HEK293T cell line was a kind gift from Prof. Maciej Kurpisz lab (Institute of Human Genetics, Polish Academy of Sciences, Poland). Cells were cultured under standard conditions in Dulbecco’s modified Eagle’s medium (Gibco, Thermo Fisher Scientific, Waltham, MA, USA) with 10% fetal bovine serum (Gibco, Thermo Fisher Scientific) and 1% penicillin/streptomycin solution (Sigma Aldrich, St. Louis, MO, USA). Six T-ALL cell lines: DND-41, CCRF-CEM, Jurkat, BE-13, P12-Ichikawa and MOLT-4, were purchased from the Leibniz Institute DSMZ—German Collection of Microorganisms and Cell Cultures. Cells were cultured under standard conditions in RPMI-1641 medium (Gibco, Thermo Fisher Scientific) with 10% fetal bovine serum (Gibco, Thermo Fisher Scientific). 

### 2.4. RNA Extraction and RT-qPCR

The miRCURY RNA Isolation Kit Cell & Plant (Qiagen, Hilden, Germany) was used for the extraction of total RNA including the recovery of the small RNA fraction. RNA isolates were DNase treated and purified with use of RNA Clean and Concentrator Kit (Zymo Research, Irvine, CA, USA). RNA concentration was measured with Quantus Fluorometer (Promega, Madison, WI, USA) using Qubit HS RNA Assay Kit (Thermo Fisher Scientific). RNA integrity was determined with 4200 Tapestation using High Sensitivity RNA ScreenTape (Agilent Technologies, Santa Clara, CA, USA) and 2100 Bioanalyzer using RNA 6000 Nano Assay (Agilent Technologies). For miRNA quantification, total RNA was reverse transcribed with TaqMan Advanced miRNA cDNA Synthesis Kit (Thermo Fisher Scientific) according to the manufacturer’s protocol. TaqMan Fast Advanced Master Mix and predesigned TaqMan Advanced miRNA assays (Thermo Fisher Scientific) were used. Three endogenous control miRNAs (hsa-miR-16-5p, hsa-let-7a-5p and hsa-miR-25-3p) were selected using a strategy based on a comprehensive assessment of expression stability in our miRNA-seq data and in RT-qPCR, as previously described [16]. For mRNA quantification, total RNA was reverse transcribed with iScript cDNA Synthesis Kit (Bio Rad, Hercules, CA, USA) and HOT FIREPol EvaGreen qPCR Mix Plus (Solis Biodyne, Tartu, Estonia) was used. Primers were synthetized by Genomed (Warsaw, Poland). List of primers used for mRNA quantification is presented in Supplementary Table S1. Geometric mean of Β-actin and GAPDH expression was used for normalization of expression of the analyzed target genes. All RT-qPCR analyses were conducted in three technical and three biological replicates (three independent, time-separated transfections) with the use of 7900HT Fast Real-Time PCR System (Applied Biosystems, Foster City, CA, USA). Comparative deltaCT method (ΔΔCT) and Data Assist Software v. 3.01 (Thermo Fisher Scientific) were used for relative quantification of expression [18]. 

### 2.5. Dual Luciferase Reporter Assay

Selected predicted miRNA-mRNA interactions were validated with Dual-Glo Luciferase Reporter Assay (Promega, Madison, WI, USA). HEK 293T cells were seeded on 24-well culture plate 24 h before transfection. Cells were subjected to transfection at 60% to 80% confluency using JetPrime DNA/siRNA Transfection Kit (Polyplus Transfection, New York, NY, USA) to enable co-transfection with relevant miRNA mimics or negative control mimics (MirVana, Thermo Fisher Scientific) and pmirGLO plasmids (Promega), containing 3′UTR fragments of the selected target genes. miRNA mimics in final concentration of 50 μM and 125 ng of plasmid were used per well. 3′UTR fragments cloned into pmirGLO plasmid contained the predicted 6-8 nt long miRNA response element (MRE), flanked with 30 nt long regions on both sides. In Dual Luciferase rescue experiments, 4 point mutations were introduced to the MRE region during the oligonucleotide synthesis step to abolish the miRNA-mRNA interaction. The list of oligonucleotides containing wild type/mutated MRE sequences with flanking sites is presented in Supplementary Table S2. Luciferase activity was measured with GloMax-Multi+ Detection System (Promega) after 48 h from transfection. All experiments were performed in three replicates. A significant decrease in luciferase activity relative to control (negative control miRNA) was indicative of direct interaction between the seed sequence of the miRNA (defined as the nucleotides at position 2–7 of the 5’ end of mature miRNA sequences) and the MRE in the 3′UTR of target mRNA. 

### 2.6. Transfection of T-ALL Cell Lines

T-ALL cell lines were transfected with negative control, hsa-miR-20b-5p and hsa-miR-363-3p mimics and inhibitors (miRVana, Thermo Fisher Scientific). Mimics and inhibitors were used in the final concentration of 50 nM and 100 nM, respectively. Cells were electroporated with the use of the Neon Electroporation System according to the manufacturer’s protocol. Briefly, cells were resuspended in Neon Resuspension R Buffer (Thermo Fisher Scientific) at a concentration of 20 × 10^6^ cells/mL and transferred to Neon Tips. Electroporation was conducted under following conditions: for DND-41 pulse voltage 1150 V, pulse width 30 ms, pulse number 2; for CCRF-CEM pulse voltage 1600 V, pulse width 20 ms, pulse number 1. 

### 2.7. Western Blotting

After transfection, 3 × 10^6^ cells per well were seeded on 6-well plate in 2 mL of culture medium. After 24, 48, and 72 h, half of the cells from each well was collected and lysed in 200 µL of RIPA buffer with protease inhibitor cocktail and EDTA (Thermo Fisher Scientific). Protein concentration was determined using Pierce BCA Protein Assay Kit (Thermo Fisher Scientific). Proteins were separated on 4%–15% Mini-PROTEAN TGX Stain-free Gel (Bio Rad). After electrophoresis, proteins were transferred onto 0.45 µm PVDF Low Fluorescence membrane (Bio Rad). Membranes were blocked using 5% non-fat milk or BSA and incubated with 1:2000 anti-PTEN (Cell Signaling Technology, #9552), 1:2000 anti-BIM (Cell Signaling Technology, Danvers, MA, USA, #2933), 1:750 anti-FBXW7 (Abcam, Cambridge, UK, AB109617) or 1:2000 anti-SOS1 (Cell Signaling Technology, #12409) antibodies. After washing, membranes were incubated with horseradish peroxidase (HRP) conjugated with 1:40,000 (for PTEN, BIM and SOS1) or 1:20,000 (for FBXW7) anti-Rabbit (Abcam, ab97051) secondary antibody. Immunoreactive protein bands were detected with Clarity Western ECL Substrate for HRP (Bio-Rad) on Chemidoc Imaging System (Bio Rad). The abundance of target protein was assessed in reference to the total protein on a blot in Stain-Free technology using Image Lab 6.0.1 software (Bio Rad). Each experiment was conducted in three technical and three biological replicates (three independent, time-separated transfections). Whole total protein membranes and chemiluminescence blots are shown in Supplementary Figure S1. 

### 2.8. AGO2-RNA Immunoprecipitation and RT-qPCR

For each immunoprecipitation reaction 3 × 10^7^ DND-41 cells were collected and crosslinked with 1% formaldehyde in PBS for 10 min in room temperature. The crosslinking was stopped by adding glycine to the final concentration of 0.25 M. Immunoprecipitation was performed with the use of Magna RIP Kit (Merck, Darmstadt, Germany). Each portion of Protein A/G magnetic beads was coated with 5 µg of either anti-Ago2 (Abnova, Taipei, Taiwan, H00027161-M01) or normal mouse IgG (Merck, CS200621) antibody. Non-RNA binding normal mouse IgG was used as negative control. Lysates from crosslinked cells were prepared according to the manufacturer’s protocol and incubated with antibody-conjugated magnetic beads in 4 °C with rotation overnight. After incubation, magnetic beads were washed and treated with proteinase K to release the RNA from ribonucleoprotein complexes. RNA was purified through Trizol isolation combined with Zymo RNA Clean and Concentrator columns. Equal volumes of immunoprecipitated mRNA and miRNA fractions from Ago2-RIP and IgG-RIP samples were reverse transcribed as described above. The abundance of hsa-miR-20b-5p, hsa-miR-363-3p, *PTEN* and *BIM* in the AGO2-RIP and IgG-RIP fraction was assessed by RT-qPCR. Fold enrichment of examined miRNAs and target mRNAs in AGO2-RIP fraction was calculated as 2-ΔCq (Cq—quantification cycle), where ΔCq = Cq(AGO2-RIP) − Cq(IgG-RIP).

### 2.9. Cell Viability Assay

After transfection, 2 × 10^4^ cells per well were seeded on 96-well plate in 100 µL of culture medium. After 0, 24, 48, 72, and 96 h, 10 µL of Cell Counting Kit 8 (Sigma Aldrich) reagent was added to each well. Cells were incubated with CCK8 reagent for 4 h in 37 °C. Absorbance was measured on the GloMax-Multi+ Detection System (Promega). Each experiment was conducted in five technical and three biological replicates (three independent, time-separated transfections). 

### 2.10. Apoptosis Assay

After transfection, 1.5 × 10^6^ cells per well were seeded on 12-well plate in 1 mL of culture medium. After 24, 48, and 72 h, half of the cells from each well were collected and stained with Annexin V PE 7-AAD Apoptosis Detection Kit (BD Biosciences, San Jose, CA, USA). Populations of apoptotic and non-apoptotic cells were detected with a FlowSight flow cytometer (Luminex Corporation, Austin, TX, USA). Results were analyzed in Ideas 6.2 Software (Luminex Corporation). Each experiment was conducted in three biological replicates (three independent, time-separated transfections). Representative apoptosis plots are shown in Supplementary Figure S2.

### 2.11. Cell Cycle Assay

After transfection 1.5 × 10^6^ cells per well were seeded on 12-well plate in 1 mL of culture medium. After 24, 48, and 72 h, half of the cells from each well was collected and fixed with methanol. Cells were then stained with propidium iodide (Sigma Aldrich). Populations of cells in G0/G1, S and G2/M phase were detected with the use of S3e Cell Sorter (Bio Rad). Results were analyzed in ModFit Software (Verity Software House, Topsham, ME, USA). Each experiment was conducted in three biological replicates (three independent, time-separated transfections). 

### 2.12. Statistical Analysis

For the comparison of two independent means, data were analyzed for normality with Shapiro–Wilk test and next the two groups were tested for equality of variances. Statistical significance of the results was calculated with unpaired two-tailed t-test or nonparametric Mann–Whitney test. For proliferation assays, statistical significance was calculated with a mixed model analysis using the Tukey post-hoc test. All analyses and data visualization were performed with GraphPad Prism 8 software.

## 3. Results

### 3.1. hsa-miR-20b-5p and hsa-miR-363-3p are Overexpressed in T-ALL and Target Genes Involved in Regulation of Apoptosis

Following our previous miRNA-seq study in pediatric T-ALL primary samples, including validation of miRNA expression by RT-qPCR in the discovery and validation cohort [12,16], we here performed additional RT-qPCR in 6 T-ALL cell lines (DND-41, CCRF-CEM, JURKAT, MOLT-4, P12-ICHIKAWA, and BE-13) to select an optimal model for in vitro experiments. We used 3 types of control cells: Normal mature T lymphocytes, CD34+ thymocyte samples, and CD4+CD8+ thymocyte samples. hsa-miR-20b-5p exhibited high level of expression in all examined T-ALL cell lines as compared to all types of control cells (Figure 1A). The expression of hsa-miR-363-3p was higher in DND-41, JURKAT, and P12-ICHIKAWA cell lines than in normal CD34+ T-cells. Yet, only DND-41 showed higher hsa-miR-363-3p expression level as compared to CD34+ and CD4+CD8+ thymocytes (Figure 1B). We confirmed overexpression of hsa-miR-20b-5p and hsa-miR-363-3p in a total cohort of T-ALL patients as compared to normal T-cells and in case of hsa-miR-20b-5p also as compared to CD4+CD8+ thymocytes (Figure 1). Of note, despite the overall overexpression of these miRNAs in T-ALL patients’ group, the expression levels were varying, which is in agreement with the considerable heterogeneity of this disease. For in vitro experiments aimed at the assessment of the phenotypic effects of hsa-miR-20b-5p and hsa-miR-363-3p mimicry and inhibition, we selected easy to transfect cell lines with varying endogenous levels of the studied miRNAs: DND-41 with high levels of both studied miRNAs and CCRF-CEM with high level of miR-20b-5p and low level of miR-363-3p, comparable to the expression levels in a subset of T-ALL patients. 

To establish the potential molecular consequences of the upregulation of the two miRNAs from cluster miR-106a-363, we used data from our bioinformatic target gene prediction and pathway enrichment analysis [12]. Genes predicted as targets for differentially expressed miRNAs by at least 5 algorithms and analyzed for enrichment in Gene Ontology (GO) and Kyoto Encyclopedia of Genes and Genomes (KEGG) terms and pathways indicated several processes of potential importance for T-ALL pathogenesis. Notably, one of the top 10 highly enriched GO terms was “Positive regulation of apoptosis” (GO:0043065). Interestingly, many target genes enriched in this term were predicted to be targeted, and thus silenced, by hsa-miR-20b-5p and hsa-miR-363-3p (Supplementary Table S3). We performed luciferase reporter assays in human embryo kidney HEK293T cell line to validate several selected interactions of hsa-miR-20b-5p and hsa-miR-363-3p with their predicted targets, as described in details in Supplementary Materials. We confirmed the majority of the predicted interactions; most of them have not been annotated in databases of validated interactions thus far.

### 3.2. hsa-miR-20b-5p and hsa-miR-363-3p Downregulate PTEN and BIM in T-ALL Cells In Vitro

We further aimed to test if these two miRNAs affect the expression of selected target genes enriched in the ‘Positive regulation of apoptotic process’ GO term. Using RT-qPCR and Western blotting, we assessed the changes in the expression of target genes after inhibition and mimicry of studied miRNAs. We focused on the interactions of hsa-miR-20b-5p and hsa-miR-363-3p with *PTEN* and *BIM*. Of note, both these genes are validated targets of both studied miRNAs, which might potentially reveal the cooperative effects of the “clustered” miRNAs on the cells’ phenotype. 

The interaction of hsa-miR-20b-5p-*PTEN* has previously been demonstrated (Supplementary Table S4) and also confirmed by us in luciferase reporter assays (Supplementary Figure S3A) [12]. hsa-miR-363-3p-*PTEN* interaction, which we validated by luciferase assay (Supplementary Figure S3B) [12], has not been previously reported by others. hsa-miR-20b-5p-*BIM* and hsa-miR-363-3p-*BIM* were not covered by our overrepresentation analysis (both were predicted by 4 and not by 5 algorithms), but have previously been reported by different approaches (Supplementary Table S4). We included these interactions in further experiments for the clear involvement of *BIM* (encoding a pro-apoptotic protein) in the positive regulation of apoptosis. Additionally, we selected for RT-qPCR and Western blotting experiments the interactions of the less evident role in apoptosis: hsa-miR-20b-*SOS1* and hsa-miR-363-*FBXW7*. For miRNA mimicry and inhibition we have used the DND-41 cell line, as the most optimal model, since it is negative for *PTEN* and *FBXW7* inactivating mutations or deletions [19] and it exhibits high endogenous expression of both studied miRNAs (Figure 1). Additionally, we used CCRF-CEM cell line, showing relatively high expression of hsa-miR-20b-5p but low expression of hsa-miR-363-3p, comparable to that in normal T-cells, but also in a subset of T-ALL patients (Figure 1). CCRF-CEM cell line is characterized by lack of *PTEN* expression due to deletion and mutation in this gene [19,20]. 

**Figure 2:**
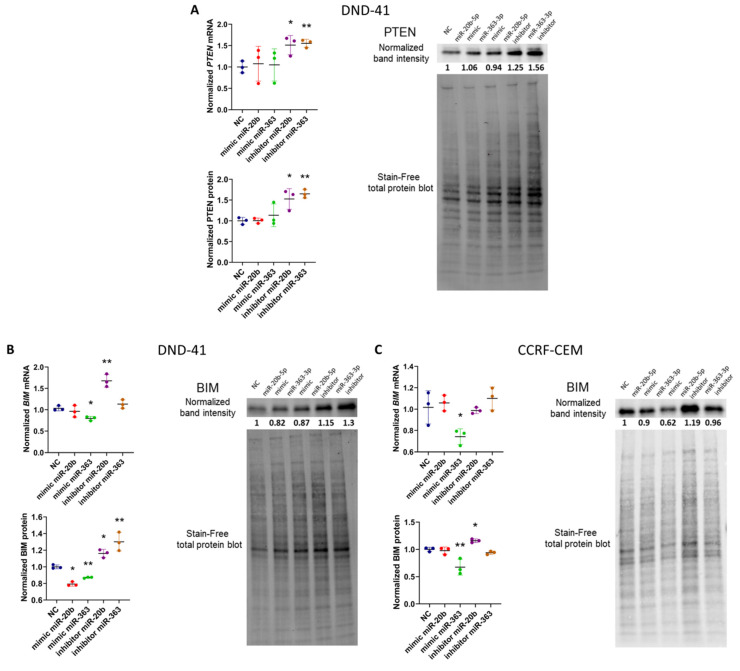
Evaluation of the expression of target genes for hsa-miR-20b-5p and hsa-miR-363-3p on mRNA and protein level after inhibition and mimicry of these miRNAs in DND-41 and CCRF-CEM T-ALL cell lines. mRNA and protein level of PTEN in DND-41 cell line (**A**), BIM in DND-41 cell line (**B**) and BIM in CCRF-CEM cell line (**C**). Scatter plots present mean values of three biological replicates; error bars indicate standard deviation. NC—negative control; ** *p* < 0.01; * *p* < 0.05.

We demonstrated that inhibition of hsa-miR-20b-5p and hsa-miR-363-3p in DND-41 cells (showing high endogenous levels of both miRNAs) resulted in statistically significant increase of *PTEN* mRNA expression (*p =* 0.028 for hsa-miR-20b-5p and *p =* 0.004 for hsa-miR-363-3p) and increase of PTEN protein level (*p =* 0.025 for hsa-miR-20b-5p and *p =* 0.0011 for hsa-miR-363-3p) (Figure 2A). We did not observe the effects of the overexpression of these miRNAs (by miRNA mimics) on *PTEN* expression, potentially due to the already high endogenous levels of these miRNAs. 

The inhibition of hsa-miR-20b-5p in DND-41 cell line resulted in an increase of *BIM* mRNA (*p =* 0.043) and BIM protein (*p =* 0.029) expression. This effect was also observed for the hsa-miR-363-3p inhibitor, with statistical significance only at the protein level (*p =* 0.009) (Figure 2B). Moreover, mimicry of hsa-miR-363-3p resulted in decrease of *BIM* mRNA expression (*p =* 0.011) and BIM protein (*p =* 0.002) (Figure 2B). While the mimicry of hsa-miR-20b-5p resulted in decrease of BIM only at the protein level (*p =* 0.024) (Figure 2B). 

In CCRF-CEM cell line we observed statistically significant effects of the hsa-miR-20b-5p inhibition on the increase of BIM protein level (*p =* 0.03). We did not observe the effects of hsa-miR-20b-5p mimicry, potentially due to the high endogenous level of this miRNA in this cell line. As expected, for the hsa-miR-363-3p (with low endogenous level in these cells) we only observed the effects of the overexpression of this miRNA on BIM expression (both at the mRNA, *p =* 0.045; and protein level, *p =* 0.019); we did not observe the effects of hsa-miR-363-3p inhibition. 

As for the miRNA interactions with the other two genes (*SOS1* and *FBXW7*) also enriched in “Positive regulation of apoptotic process” GO term, but with less obvious functional link to this process, the effects of miRNA inhibition and mimicry were less convincing. In DND-41 cells, the mimicry of hsa-miR-20b-5p resulted in statistically significant decrease of *SOS1* mRNA (*p =* 0.035) and protein (*p =* 0.03) level; inhibition resulted in increase of protein expression (*p =* 0.042) (Supplementary Figure S4A) Yet, such effects were not observed in CCRF-CEM cells (Supplementary Figure S4B). In case of *FBXW7*, mimicry of hsa-miR-363-3p resulted in decreased mRNA level in DND-41 cell, yet these changes were not statistically significant (a trend was observed; *p =* 0.059) and were not reflected at protein level. We did not observe any significant effects of miRNA mimicry and inhibition in the CCRF-CEM cell line (Supplementary Figure S4C,D). For these reasons, we excluded these miRNA-mRNA interactions from further experiments.

Thus, by a combined use of miRNA mimics and inhibitors, we demonstrated that hsa-miR-20b-5p and hsa-miR-363-3p both, to various extend, regulate the expression of *PTEN* and *BIM* tumor suppressor genes in T-ALL cells in vitro. To provide additional evidence for these two miRNAs exerting posttranscriptional silencing of *PTEN* and *BIM* in T-ALL cells, we performed RNA immunoprecipitation (RIP) in non-transfected DND-41 cells with the use of an antibody against AGO2. AGO2 is the component of RNA-induced silencing complex (RISC). AGO2-RIP followed by RT-qPCR analysis of immunoprecipitated RNA enabled to demonstrate the enrichment of hsa-miR-20b-5p, hsa-miR-363-3p, *PTEN* and *BIM* in AGO2-RIP fraction as compared to control IgG-RIP fraction (immunoprecipitation with use of normal mouse IgG) (Supplementary Figure S5). Thereby, we showed that both these miRNAs and both their target mRNAs interact in RISC complexes in T-ALL cells.

### 3.3. hsa-miR-20b-5p and hsa-miR-363-3p Exert Anti-Apoptotic and Pro-Proliferative Effects in T-ALL Cells In Vitro

**Figure 3:**
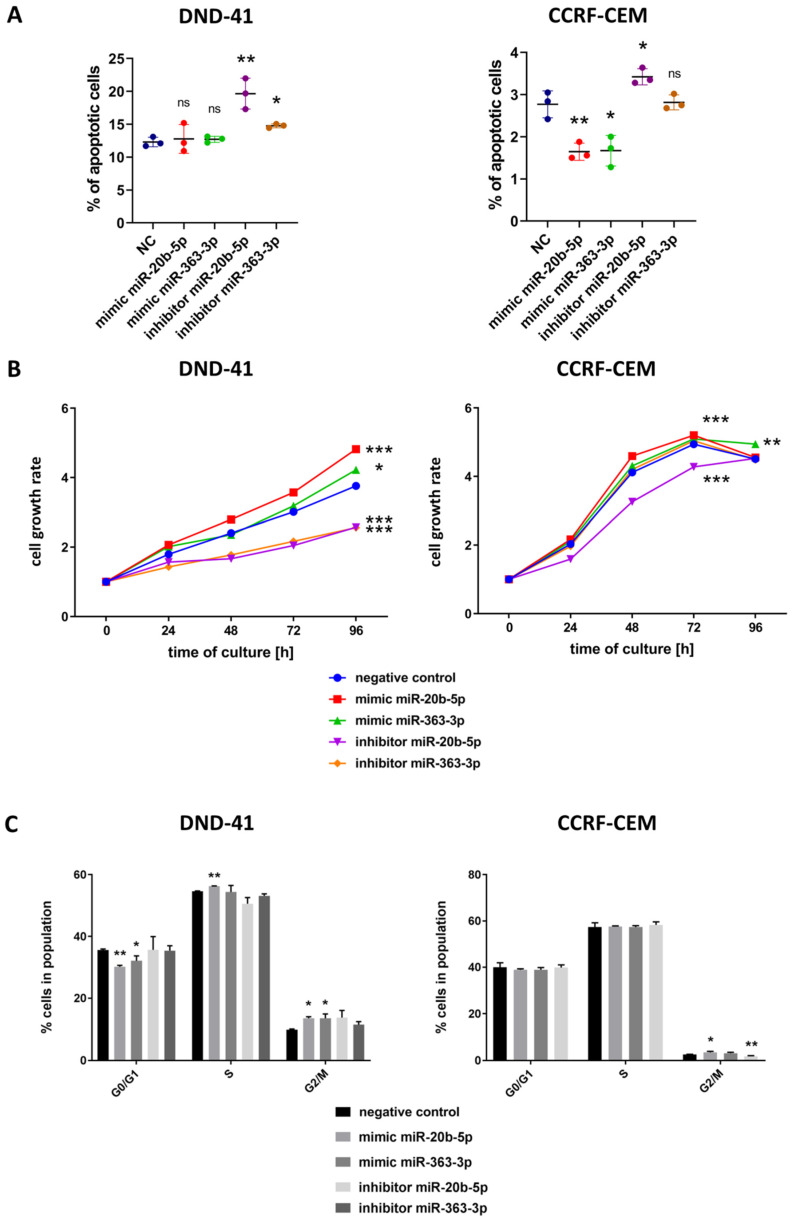
Functional effect of inhibition and mimicry of hsa-miR-20b-5p and hsa-miR-363-3p on apoptosis (**A**), cell growth measured by colorimetric viability assay (**B**) and cell cycle distribution (**C**) of DND-41 and CCRF-CEM T-ALL cell lines. Cell growth rate (**B**) was calculated as fold change of OD450 for each examined time point in reference to the starting point (0 h). Scatter plots and bar plots present mean values of three biological replicates; error bars indicate standard deviation. *** *p* < 0.001; ** *p* < 0.01; * *p* < 0.05.

Finally, we assessed the phenotypic effects of the downregulation and upregulation of hsa-miR-20b-5p and hsa-miR-363-3p on the apoptosis, viability and cell cycle of DND-41 and CCRF-CEM cell lines. We transfected T-ALL cells with the mimics and inhibitors of the studied miRNAs and performed flow cytometry analysis of apoptosis. Inhibition of hsa-miR-20b-5p and hsa-miR-363-3p in DND-41 cells resulted in increased number of apoptotic cells in comparison to negative control (*p =* 0.0065 for hsa-miR-20b-5p and *p =* 0.025 for hsa-miR-363-3p) indicating stimulation of spontaneous apoptosis of the cells upon downregulation of these miRNAs (Figure 3A). Of note, the mimicry of these miRNAs resulted in no significant changes in the number of apoptotic DND-41 cells, which was in line with the results of mRNA and protein expression analysis—statistically significant effects were mainly observed for the inhibitors, and less for the mimics (Figure 2A,B). In CCRF-CEM cell line the inhibition of hsa-miR-20b-5p resulted in enhanced apoptosis of the cells *p =* 0.038), while overexpression of hsa-miR-20b-5p and also of hsa-miR-363-3p, by the use of miRNA mimics, caused reduction in the number of apoptotic cells as compared to controls (*p =* 0.006 for hsa-miR-20b-5p and *p =* 0.017 for hsa-miR-363-3p) (Figure 3A). These confirmed our observation from the DND-41 cell line on the enhanced apoptosis upon downregulation of these miRNAs and additionally demonstrated resistance to spontaneous apoptosis upon upregulation of these miRNAs. These results also largely reflect the pattern of the effects of miRNA inhibition and mimicry in mRNA and protein expression analysis (Figure 2C). This data implies that upregulated levels of hsa-miR-20b-5p and hsa-miR-363-3p in T-ALL cells protect them from apoptosis and thus might promote their survival.

In the next step, we evaluated the effects of miRNA mimicry and inhibition on the survival of T-ALL cells in vitro. In both cell lines, we observed statistically significant increase in the viability of T-ALL cells after overexpression of hsa-miR-20b-5p (*p <* 0.0001 for DND-41 and also for CCRF-CEM) and reduction of viability after hsa-miR-20b-5p inhibition (*p <* 0.0001 for both cell lines) (Figure 3B). Introduction of hsa-miR-363-3p mimics to DND-41 cells had moderate effect (*p =* 0.0121), but inhibition of this miRNA resulted in significant reduction of viability (*p <* 0.0001). Moreover, we observed enhanced viability of CCRF-CEM cells upon overexpression of hsa-miR-363-3p (*p =* 0.0036). We did not observe statistically significant effects of hsa-miR-363-3p inhibition in CCRF-CEM cells (Figure 3B), potentially due to low endogenous level of this miRNA in these cells. This observation was in line with no effects of hsa-miR-363-3p inhibition on mRNA nor protein level of BIM in these cells (Figure 2C). For these reasons, we did not test this variant (hsa-miR-363-3p inhibition) in further experiments in this cell line. 

Interestingly, despite increased survival of DND-41 cells upon mimicry of hsa-miR-20b-5p and hsa-miR-363-3p (Figure 3B), we observed no differences in the apoptosis of these cells (Figure 3A). Thus, we hypothesized that the higher viability of DND-41 cells upon miRNA mimicry is caused by enhanced proliferation rather than suppressed apoptosis. To test this, we performed flow cytometry assay for cell cycle distribution to assess the percentage of non-dividing and dividing cells. In DND-41 cells, mimicry of hsa-miR-20b-5p and hsa-miR-363-3p resulted in decreased number of the cells in G0/G1 phase (*p =* 0.006 for hsa-miR-20b-5p and *p =* 0.048 for hsa-miR-363-3p) and increased number of cells in G2/M (*p =* 0.014 for hsa-miR-20b-5p and *p =* 0.036 for hsa-miR-363-3p) and S phase (*p =* 0.0022 for hsa-miR-20b-5p) (Figure 3C), thus confirming our assumption on the enhanced proliferation rate of T-ALL cells upon overexpression of these miRNAs. In CCRF-CEM cells, the effects were less profound, overexpression of hsa-miR-20b-5p resulted in an increased number of proliferating cells (*p =* 0.032 for cells in G2/M phase as compared to negative control), while inhibition of this miRNA gave the opposite effect (*p =* 0.0092) (Figure 3C). We observed no statistically significant differences in cell cycle distribution in CCFR-CEM cell line upon overexpression of hsa-miR-363-3p. 

**Figure 4:**
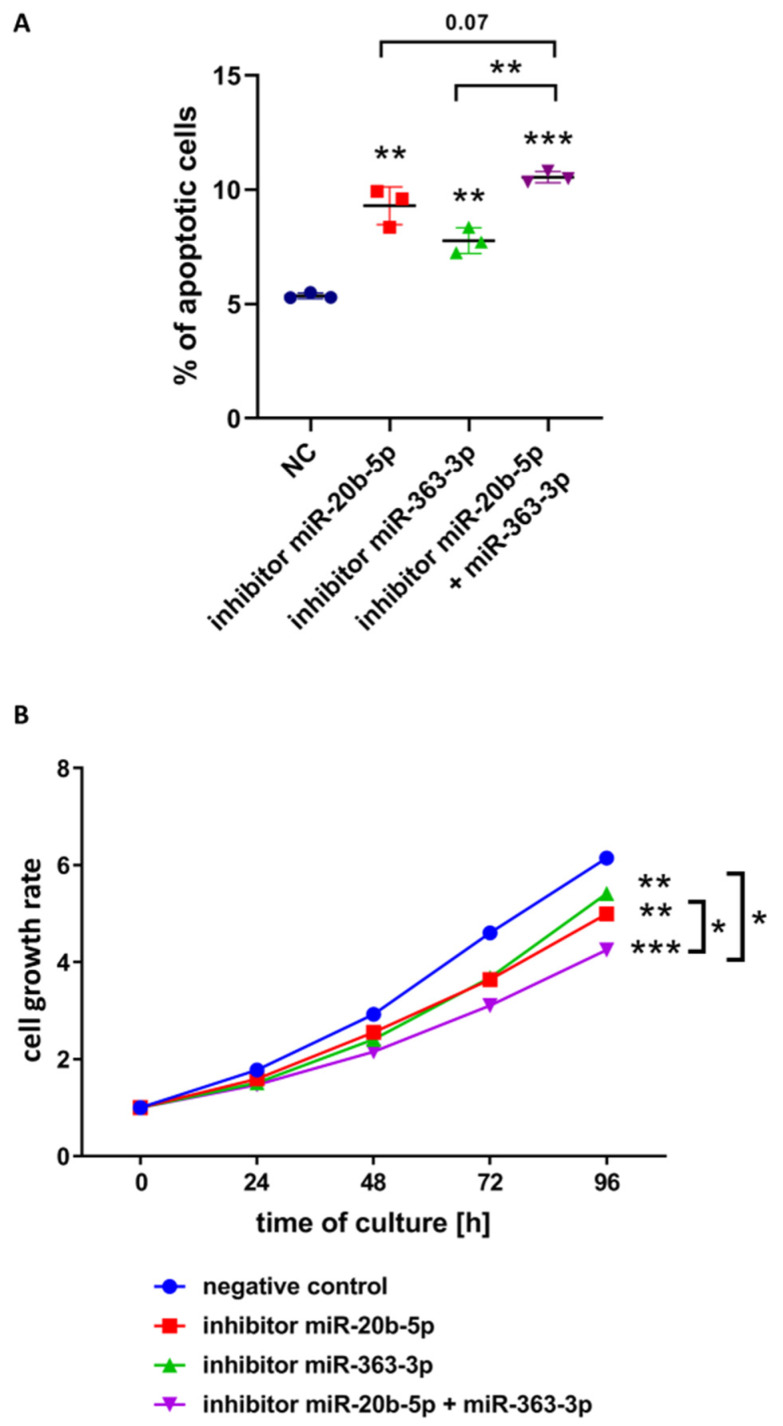
Functional effect of simultaneous inhibition of hsa-miR-20b-5p and hsa-miR-363-3p as compared to negative control and to inhibition of single miRNAs in DND-41 T-ALL cell line. (**A**) Percentage of apoptotic cells in all examined variants. (**B**) Cell growth rate, measured by colorimetric viability assay and calculated as fold change of OD450 for each examined time point in reference to the starting point (0 h). Asterisks without brackets indicate the statistical significance of differences between negative control and each examined miRNA inhibition variant. Scatter plot presents mean values of three biological replicates; error bars indicate standard deviation. *** *p <* 0.001; ** *p <* 0.01; * *p <* 0.05.

We further hypothesized that the functional effects mediated by the cooperative action of these two miRNAs are greater than the effect of a single miRNA. We performed another set of apoptosis, growth and cell cycle assays to compare the effects of the simultaneous inhibition of both clustered miRNAs versus inhibition of single miRNAs. To this aim we chose DND-41 cell line, since both studied miRNAs are highly expressed in these cells (Figure 1) and both, *PTEN* and *BIM* are expressed (Figure 2A,B). Indeed, the concurrent inhibition of both miRNAs resulted in higher percentage of apoptotic cells in DND-41 culture than in culture variants with single miRNA inhibitors (*p =* 0.0014 for simultaneous inhibition vs. hsa-miR-363-3p inhibition; *p =* 0.07 for simultaneous inhibition vs. hsa-miR-20b-5p inhibition) (Figure 4A). This observation was in line with the decreased growth of cells transfected by both inhibitors as compared to negative control (*p <* 0.0001) and as compared to cells transfected with single inhibitors (*p =* 0.0143 for hsa-miR-363-3p inhibition; *p =* 0.0234 for hsa-miR-20b-5p inhibition) (Figure 4B). As earlier, we observed no clear differences in cell cycle distribution after inhibition of single miRNAs (Figure 3C) but simultaneous inhibition of both miRNAs resulted in slight increase of percentage of cells in G0/G1 phase as compared to the control (*p =* 0.043) (Supplementary Figure S6).

## 4. Discussion

Here we aimed to investigate if hsa-miR-20b-5p and hsa-miR-363-3p act as oncogenic miRNAs in T-ALL cells in vitro. We selected these miRNAs since they belong to one cluster of miRNAs (mir-106a-363), showed overexpression in a substantial number of primary T-ALL samples in our previous miRNA-seq study and both potentially silence genes involved in positive regulation of apoptosis (Supplementary Table S3) [12]. Some of these genes, like *PTEN*, *FBXW7,* or *BIM*, have already established tumor suppressive and pro-apoptotic roles [6,21,22,23], yet their interactions with hsa-miR-20b-5p and hsa-miR-363-3p have not been studied functionally in T-ALL thus far. We aimed to test, if silencing of these genes by upregulated hsa-miR-20b-5p and hsa-miR-363-3p may contribute to oncogenic mechanism in T-ALL cells in vitro. 

PTEN is a negative regulator of PI3K/AKT pathway and thus is involved in the inhibition of cell growth and proliferation and in the promotion of apoptosis [22]. *PTEN* is affected by deletions and/or inactivating mutations in many types of malignancies, including T-ALL, contributing to oncogenic activation of PI3K-AKT pathway [20,22,24,25]. In pediatric T-ALL, *PTEN* abnormalities are found in approx. 15% of pediatric T-ALL cases and have been shown, also by our group, to be related to therapy resistance and poor prognosis [26,27,28,29]. *BIM* (alias *BCL2L11*) encodes a protein from BCL-2 family of apoptosis-related proteins acting as apoptotic activator [21]. *BIM* has been demonstrated to be repressed by upregulated *MYC* and *PI3K-AKT* pathways resulting in enhanced survival of T-ALL cells [30]. Mutations of *BIM* have been shown to induce resistance to apoptosis despite downregulation of *MYC* in T-ALL zebrafish model [31]. *FBXW7* is involved in ubiquitination and degradation of *MYC* and *NOTCH1* [32]. While *MYC* is a “pan-cancer” oncogene [33], *NOTCH1* is more “T-ALL-specific”. It acts as a ligand-activated transcription factor, critical for the differentiation and development of early T-cell precursors in the thymus [34]. Inactivating mutations of *FBXW7* (identified in approx. 30% of T-ALL cases and frequently co-occurring with activating *NOTCH1* mutations) result in decreased degradation of activated NOTCH1 [5]. We additionally investigated *SOS1* gene, which role in T-ALL is much less understood. *SOS1* is involved in the activation of RAS pathway [35,36], thus it is more likely to be involved in the negative, and not positive, regulation of apoptosis. *SOS1* was also shown to be crucial for early T-cell development; its knockout in mice resulted in differentiation blockade of thymocytes [37]. 

To test if hsa-miR-20b-5p and hsa-miR-363-3p cause silencing of *PTEN*, *BIM*, *FBXW7,* and *SOS1*, we applied miRNA mimicry and inhibition in T-ALL cells in vitro. We selected two easy to transfect T-ALL cell lines with varying endogenous levels of the studied miRNAs: DND-41 with high levels of both studied miRNAs and CCRF-CEM with high level of miR-20b-5p and low level of miR-363-3p. The choice was aimed to reflect the heterogeneity of the disease, observed also at the miRNA transcriptome level [12].

Since in humans and animals, the mechanism of miRNA-mediated regulation of gene expression relies more on blocking of translation rather than mRNA degradation, we assessed mRNA and protein expression. We demonstrated regulatory effects of hsa-miR-20b-5p and hsa-miR-363-3p over *BIM* (in both T-ALL cell lines) and *PTEN* (in DND-41 cells). Targeting of *BIM* and *PTEN* by hsa-miR-20b-5p and hsa-miR-363-3p was reflected by decreased levels of proteins upon miRNA mimicry and by the reverse effects upon miRNA inhibition; in some cases the effects were also seen at the mRNA level (Figure 2). Of note, these regulatory effects were not observed in every variant of miRNA inhibition and mimicry and varied in their statistical significance. This is not surprising, considering the mode of action of miRNAs and thus usually mild effects of a single miRNA on the expression of a single target gene [2,38,39]. What is more, the endogenous levels of the studied miRNAs differed in the studied cell lines, which we considered in the interpretation of our results. Finally, the more profound effects, which we observed in case of miRNA inhibitors than miRNA mimics, might also reflect the different modes of action of these two types of molecules related to their different structure [40]. Despite these imperfections of in vitro conditions, we demonstrated, either by the effects of miRNA inhibition or miRNA mimicry, or both, that miR-20b-5p and miR-363-3p affect the levels of *PTEN* and *BIM* genes, proteins, or both. Our immunoprecipitation results (Supplementary Figure S5) are also indicative of the interaction of both miRNAs with *PTEN* and *BIM* in T-ALL cells in vitro and support our conclusions from RT-qPCR and Western blotting on the regulatory roles of these miRNAs over these genes. 

Interestingly, despite the existence of two potential binding sites in *FBXW7* 3’UTR for hsa-miR-363-3p [41,42] we found no statistically significant evidence for this regulation in the examined T-ALL cell lines. As for *SOS1*, being a putative target of hsa-miR-20b-5p, we demonstrated the regulatory effect only in DND-41 cells (Supplementary Figure S4).

To further test our hypothesis, that hsa-miR-20b-5p and hsa-miR-363-3p act as oncogenes in T-ALL cells in vitro, we investigated the effects of miRNA inhibition and mimicry on apoptosis, growth and cell cycle in DND-41 and CCRF-CEM cell lines. Altogether, our results show that hsa-miR-20b-5p and hsa-miR-363-3p, to various extent, affect the growth of T-ALL cells by inhibiting spontaneous apoptosis and by stimulating the proliferation, upon overexpression of these miRNAs, and by the reverse effects (pro-apoptotic and anti-proliferative) after the inhibition of these miRNAs. Our results point to potentially important roles of *PTEN* and *BIM* as mediators of these effects.

Of note, the functional effects of miRNAs inhibition and mimicry were more profound in DND-41 cells than in CCRF-CEM cells. The interpretation of these observations should account for the endogenous expression of the miRNAs of interest and their targets: DND-41 cells express both miRNAs at high levels as compared to T-ALL primary samples and all studied controls, and express both *PTEN* and *BIM*; CCRF-CEM cells express high level of hsa-miR-20b-5p, but relatively lower level of hsa-miR-363-3p, and lack *PTEN* expression. Although the low endogenous level of hsa-miR-363-3p might itself explain the less profound effects observed in these cells, it is also tempting to speculate that the survival advantage of T-ALL cells is higher, when mediated by the interactions of hsa-miR-20b-5p and hsa-miR-363-3p with both tumor suppressor genes, *PTEN* and *BIM*. Hypothetically, overexpression of these oncogenic miRNAs might serve as an alternative oncogenic mechanism to deletions and inactivating mutations of *PTEN* and contribute to loss or diminished activity of this important tumor suppressor in T-ALL cells expressing wild type *PTEN*. Naturally, other genes might also be the mediators of the impact of these miRNAs on T-ALL cells’ phenotype.

What is more, by demonstrating enhanced effects of simultaneous inhibition of hsa-miR-20b-5p and hsa-miR-363-3p in DND-41 cells as compared to single inhibitors, we point to the cooperating oncogenic effects of both miRNAs. The impact of simultaneous inhibition on the increasing number of apoptotic cells, and decreasing growth rate was less evident as compared to hsa-miR-20b-5p inhibition than as compared hsa-miR-363-3p inhibition. This might suggest the leading regulatory role of hsa-miR-20b-5p over the cells’ growth and the cooperative role of hsa-miR-363-3p. It is tempting to speculate, that overexpression of miRNAs from mir-106a-363 cluster might serve as an alternative oncogenic mechanism to overexpression of miRNAs from a prototypic oncogenic cluster mir-17-92 [43,44]. 

mir-106a-363 cluster is one of the two highly conserved paralogs of cluster mir-17-92; the second paralog is mir-106b-25 [44,45]. mir-106a-363, although much less thoroughly examined than mir-17-92, has been demonstrated to act in both oncogenic and tumor suppressor manner in various malignancies [46,47,48,49]. Since functional effects mediated by miRNAs are highly context-dependent (tissue-, disease-, time-specific), miRNAs having oncogenic activities in one type of cancer may act as tumor suppressors in other malignancies. hsa-miR-20b and hsa-miR-363-5p were shown to be downregulated and to have anti-proliferative effects in oral squamous carcinoma cells in vitro [49]. On the other hand, in an in vitro model of Ewing sarcoma, hsa-miR-20b, hsa-miR-106a, and hsa-miR-18b were shown to be upregulated and to promote cell growth [46]. All three paralogs were proven to act as oncogenes in several types of brain tumors, as reviewed by Gruszka et al. [47]. hsa-miR-20b-3p, hsa-miR-20a-5p and hsa-miR-106a-5p were shown to be upregulated and negatively correlated with *PTEN* expression in primary cutaneous B-cell lymphoma patients [48]. As regards the T-lymphoid malignancies, the most extensively studied was mir-17-92 cluster, and particularly miR-19, which was thoroughly analyzed using in vitro and in vivo T-ALL models by Mavrakis et al. [15,50]. The authors revealed a set of tumor suppressor genes (including *PTEN*, *BIM,* and *FBXW7*) negatively regulated by miRNAs from cluster mir-17-92 and suggested the potential oncogenic roles of other paralogous clusters, yet the functional evidence for their contribution to T-ALL biology was not provided in their study. 

The potential role of mir-106a-363 cluster in T-cell malignancies has already been approached in 2005 and 2007 by Landais et al., using murine model [51,52]. The authors showed that the locus referred to as Kis2 (now known to be the locus of mir-106a-363 cluster) is a frequent integration site for murine leukemia retroviruses in mouse retrovirus-induced T-cell lymphoma model. This observation was confirmed by Lum et al., who demonstrated that this locus is frequently affected by retroviral integration resulting in T-lymphomagenesis in mice. The authors have shown higher expression of murine primary mmu-mir-106a-363 transcript as well as mature mmu-miR-106a and mmu-miR-363 in tumors with retroviral integration site within this locus as compared to tumors with other integration sites [53]. In another study of Landais et al., they showed higher expression of mmu-miR-20b, mmu-miR-106a, mmu-miR-19b-2 and mmu-miR-92-2 in murine retrovirus-induced T-cell lymphomas with retroviral integration within mir-106a-363 locus as compared to lymphomas without retroviral integration in this locus [52]. The authors additionally demonstrated overexpression of human primary mir-106a-363 transcript in 9 human primary T-ALL samples as compared to normal mononuclear cells from peripheral blood and suggested their oncogenic potential. Yet, lack of functional evidence from human T-ALL cell lines or mouse models and low number of studied T-ALL patients limited the strength of this conclusion. In 2017, Kuppers et al. demonstrated that the activation of mir-106a-363 locus together with loss of p27Kip1 (tumor suppressor involved in regulation of cell cycle) leads to T-lymphomagenesis in murine model [54]. All these findings suggest that miRNAs encoded within this cluster may be implicated in T-cell lymphomagenesis [51,52,53,54]. Despite these advances in the understanding of the potential role of mir-106a-363 cluster in T-cell malignancies, no functional studies in human T-ALL cells have been conducted so far. Thus, our results fill this research gap and provide evidence for the potential involvement of miRNAs from mir-106a-363 cluster as oncomiRs in human T-ALL cells. 

### Limitations and Future Directions

In the present study we used a transient transfection approach, which results in relatively short duration of mimics and inhibitors in the cells and consequently time-restricted effects of miRNA upregulation and downregulation [55]. The effects observed in the examined T-ALL cell lines, although consistent, could be more profound with the use of stable miRNA expression approach (e.g., lentiviral transduction system). We are also aware of the limitations of the selection of target genes for the miRNAs of interest. Our target prediction was based on several prediction algorithms, yet only a fraction of multiple hypotheses generated by bioinformatics approaches is usually tested in functional experiments. Our results show that the oncogenic effects of hsa-miR-20b-5p and hsa-miR-363-3p in T-ALL cells in vitro might be mediated by the downregulation of *PTEN* and *BIM*. Obviously, other target genes might also contribute to the observed effects. The future direction of our research, is the investigation of the miRNA targetome, using AGO2 RNA immunoprecipitation combined with next generation sequencing, which allows to investigate miRNA-mediated gene regulation in a global manner [13,56]. 

Our findings on the potential oncogenic roles of hsa-miR-20b-5p and hsa-miR-363-3p in T-ALL cells can further be developed into a clinically relevant direction. This could include the integration of miRNA-seq and mRNA-seq data from T-ALL patients’ samples to search for anti-correlation patterns of expression of these miRNAs and *PTEN*, *BIM,* and presumably other genes implicated in miRNA-mediated regulation of leukemic cells’ survival. In this perspective, it might also be informative to test the effects of miRNA mimics and inhibitors in human primary T-ALL cells to verify our observations in T-ALL cell lines. Finally, our results bring to mind the perspective of testing the hypothetical therapeutic potential of antagonists of tumorigenic miRNAs, which might potentially prove beneficial for T-ALL patients, when combined with standard therapy or other therapeutic options [57,58,59]. Naturally, the potential benefits of such therapeutics would be limited to a well-defined subgroup of patients (with overexpression of hsa-miR-20b-5p and hsa-miR-363-3p), which falls into the idea of theragnostics and personalized targeted therapy in cancer [60].

## 5. Conclusions

Our study is the first to demonstrate anti-apoptotic and pro-proliferative effects of the overexpression of hsa-miR-20b-5p and hsa-miR-363-3p in human T-ALL cells in vitro. We conclude that these oncogenic effects might be mediated by the downregulation of *PTEN* and *BIM*, two important tumor suppressor genes. We postulate that these two miRNAs from mir-106a-363 cluster, when overexpressed in T-ALL cells, may act as oncomiRs by affecting the regulation of apoptosis and by favoring the proliferation, thus contributing to enhanced survival of leukemic cells. 

## Supplementary Materials

The following are available online at https://www.mdpi.com/2073-4409/9/5/1137/s1, SUMPLEMENTARY MATERIALS, Figure S1: Whole total protein membranes and chemiluminescence blots. Figure S2: Representative apoptosis plots for DND-41 (A) and CCRF-CEM (B) cell lines after inhibition or mimicry of hsa-miR-20b-5p and hsa-miR-363-3p. Figure S3: Validation of interaction between studied miRNAs and their predicted target 3′UTRs in HEK293T cell line via Dual Luciferase Reporter Assay. Figure S4: Evaluation of the expression of target genes for hsa-miR-20b-5p and hsa-miR-363-3p on mRNA and protein level after inhibition and mimicry of these miRNAs in DND-41 and CCRF-CEM T-ALL cell lines. Figure S5: Fold enrichment of hsa-miR-20b-5p, hsa-miR-363-3p, *PTEN* and *BIM* in AGO2-RIP fraction in reference to IgG-RIP (negative control) fraction. Figure S6: Functional effect of simultaneous inhibition of hsa-miR-20b-5p and hsa-miR-363-3p as compared to negative control and to inhibition of single miRNAs on cell cycle distribution in DND-41 T-ALL cell line. Table S1: List of primers used for RT-qPCR. Table S2: List of oligonucleotides used for cloning into pmiRGLO plasmid. Table S3: miRNA-mRNA interactions for genes overrepresented in positive regulation of apoptosis GO term. Table S4: miRNA-3’UTR interactions of hsa-miR-20b-5p and hsa-miR-363-3p potentially involved in positive regulation of apoptosis.
