# Supplementary material for: hsa-miR-20b-5p and hsa-miR-363-3p Affect Expression of PTEN and BIM Tumor Suppressor Genes and Modulate Survival of T-ALL Cells In Vitro
Source: Cells. 2020 May 5;9(5):1137. doi: 10.3390/cells9051137 (PMC7290785; doi:10.3390/cells9051137)
Supplement: Supplementary file 1 [file cells-09-01137-s001.zip › Suplementary materials/2020-03-14 Drobna et al SUPPLEMENTARY MATERIALS.docx]

# SUPPLEMENTARY MATERIALS

hsa-miR-20b-5p and hsa-miR-363-3p affect expression of *PTEN* and *BIM* tumor suppressor genes and modulate survival of T-ALL cells *in vitro*

Monika Drobna, Bronisława Szarzyńska, Roman Jaksik, Łukasz Sędek, Anna Kuchmiy, Tom Taghon, Pieter Van Vlierberghe, Tomasz Szczepański, Michał Witt, Małgorzata Dawidowska

## Validation of predicted miRNA-mRNA interactions by luciferase reporter assays

miRNA-mRNA interactions were selected from our previous miRNA-seq study and bioinformatic target gene prediction and pathway enrichment analysis. Genes predicted as targets for differentially expressed miRNAs by at least 5 algorithms and analyzed for enrichment in Gene Ontology (GO) and Kyoto Encyclopedia of Genes and Genomes (KEGG) terms and pathways indicated several processes of potential importance for T-ALL pathogenesis. One of the top 10 highly enriched GO terms was ‘Positive regulation of apoptosis’. Interestingly, many target genes enriched in this term were predicted to be targeted, and thus silenced, by hsa-miR-20b-5p and hsa-miR-363-3p [Supplementary Table 3]. Some of them (*PTEN* and *FBXW7*) are already known as tumor suppressors in T-ALL, yet not extensively studied in terms of their regulation by the miRNAs of interest. Several of miRNA-mRNA interactions from our list (miR-20b-*PTEN*, miR-20b-*TXNIP*, miR-20b-*UBC*, and hsa-miR-363-3p-*FNIP1*) have already been experimentally confirmed, yet have not been studied for their potential involvement in T-ALL pathogenesis. Others have not been validated thus far, nor have been studied functionally in the context of T-ALL.

Thus, we performed luciferase reporter assays in human embryo kidney HEK293T cell line to validate selected miRNA-mRNA interactions. Cells were co-transfected with dual luciferase vectors containing fragments of 3’ untranslated region (3’UTR) of potential target genes, together with hsa-miR-20b-5p mimic or hsa-miR-363-3p mimic or with negative control (‘scrambled’ miRNA). Significant downregulation of luciferase activity in the presence of miRNA mimic as compared to negative control was observed for the majority of the analyzed miRNAs-mRNAs [Supplementary Fig. 2], which was indicative of direct interaction of miRNAs and 3’UTRs of their target genes. Luciferase activity was restored by introduction of mutations in miRNA response element (MRE) region in 3’UTR fragments [Supplementary Fig. 2]. With this approach we confirmed 8/11 of the predicted interactions, namely: hsa-miR-363-3p-*LATS2*, hsa-miR-363-3p-*PTEN*, miR-20b-*PTEN*, miR-20b-*SOS1*, hsa-miR-363-3p-*FBXW7*, hsa-miR-363-3p-*NOX4*, and hsa-miR-363-3p-*NSMAF.* Most of these interactions, which we here demonstrated, have not been annotated in databases of validated interactions thus far [Supplementary Table 4].
