# Supplementary material for: hsa-miR-20b-5p and hsa-miR-363-3p Affect Expression of PTEN and BIM Tumor Suppressor Genes and Modulate Survival of T-ALL Cells In Vitro
Source: Cells. 2020 May 5;9(5):1137. doi: 10.3390/cells9051137 (PMC7290785; doi:10.3390/cells9051137)
Supplement: Supplementary file 1 [file cells-09-01137-s001.zip › Suplementary materials/2020-03-14 Drobna et al. Supplementary Table 1.docx]

**Supplementary Table 1.** List of primers used for RT-qPCR

| **Gene ID** | **Forward primer** | **Reverse primer** |
| --- | --- | --- |
| **Endogenous normalizer genes** | | |
| *ACTB* | CTTCCTGGGCATGGAGTCC | ATCTTGATCTTCATTGTGCTG |
| *GAPDH* | TGGTCACCAAGGGCTGCTT | AGCTTCCCGTTCTCAGCCTT |
| **Target genes** | | |
| *BIM (BCL2L11)* | CCACCACTTGATTCTTGCAG | GTTGCTTTGCCATTTGGTCT |
| *PTEN* | ACCCACCACAGCTAGAACTT | GGGAATAGTTACTCCCTTTTTGTC |
| *FBXW7* | TGCAAGTGATAGAACCCCAG | TGTCTTCAGCCAAAATTCTCC |
| *SOS1* | AGGAAGCGAAGAAACCCTTT | GTCAGCACACATTGCCACTT |
