# Supplementary material for: hsa-miR-20b-5p and hsa-miR-363-3p Affect Expression of PTEN and BIM Tumor Suppressor Genes and Modulate Survival of T-ALL Cells In Vitro
Source: Cells. 2020 May 5;9(5):1137. doi: 10.3390/cells9051137 (PMC7290785; doi:10.3390/cells9051137)
Supplement: Supplementary file 1 [file cells-09-01137-s001.zip › Suplementary materials/2020-03-14 Drobna et al. Supplementary Table 4.docx]

**Supplementary Table 4.** miRNA-3’UTR interactions of hsa-miR-20b-5p and hsa-miR-363-3p potentially involved in positive regulation of apoptosis

| **miRNA ID** | **Target gene** | **Number of predicted MREs** | **Annotated in databases of validated miRNA-mRNA interactions  database/ validation method** | **Validated experimentally by our group validation method** |
| --- | --- | --- | --- | --- |
| hsa-miR-20b-5p | *BIM (BCL2L11)* | 1 | miRTarBase [1] / PAR-CLIP, HITS-CLIP [2–5] | RT-qPCR, Western Blot [present study Fig. 2] |
| hsa-miR-20b-5p | *PTEN* | 1 | miRTarBase [1] / Luciferase Assay, RT-qPCR [6,7], HITS-CLIP [8] | Luciferase Assay [9], RT-qPCR, Western Blot [present study Fig. 2] |
| hsa-miR-20b-5p | *SOS1* | 1 | - | Luciferase Assay [9] |
| hsa-miR-20b-5p | *FGD4* | 2 | - | Luciferase Assay - lack of interaction [present study Supplementary Fig. 1] |
| hsa-miR-20b-5p | *TXNIP* | 1 | miRTarBase [1] / PAR-CLIP [2,10], HITS-CLIP [8] | - |
| hsa-miR-363-3p | *BIM (BCL2L11)* | 1 | miRTarBase [1] / Luciferase Assay, RT-qPCR, Immunoblot [11], HITS-CLIP, PAR-CLIP [2–5] | - |
| hsa-miR-363-3p | *PTEN* | 1 | - | Luciferase Assay [9], RT-qPCR, Western Blot [present study Fig. 2] |
| hsa-miR-363-3p | *FBXW7* | 2 | - | Luciferase Assay [present study Supplementary Fig. 1] |
| hsa-miR-363-3p | *NOX4* | 2 | - | Luciferase Assay [present study Supplementary Fig. 1] |
| hsa-miR-363-3p | *NSMAF* | 1 | - | Luciferase Assay [present study Supplementary Fig. 1] |
| hsa-miR-363-3p | *LATS2* | 1 | - | Luciferase Assay [9] |
| hsa-miR-363-3p | *FNIP* | 2 | miRTarBase [1] / PAR-CLIP [3] | - |

**References**

[1] Chou C-H, Shrestha S, Yang C-D, Chang N-W, Lin Y-L, Liao K-W, et al. miRTarBase update 2018: a resource for experimentally validated microRNA-target interactions. Nucleic Acids Res 2018;46:D296–302. doi:10.1093/nar/gkx1067.

[2] Hafner M, Landthaler M, Burger L, Khorshid M, Hausser J, Berninger P, et al. Transcriptome-wide identification of RNA-binding protein and microRNA target sites by PAR-CLIP. Cell 2010;141:129–41. doi:10.1016/j.cell.2010.03.009.

[3] Kishore S, Jaskiewicz L, Burger L, Hausser J, Khorshid M, Zavolan M. A quantitative analysis of CLIP methods for identifying binding sites of RNA-binding proteins. Nat Methods 2011;8:559–64. doi:10.1038/nmeth.1608.

[4] Memczak S, Jens M, Elefsinioti A, Torti F, Krueger J, Rybak A, et al. Circular RNAs are a large class of animal RNAs with regulatory potency. Nature 2013;495:333–8. doi:10.1038/nature11928.

[5] Whisnant AW, Bogerd HP, Flores O, Ho P, Powers JG, Sharova N, et al. In-depth analysis of the interaction of HIV-1 with cellular microRNA biogenesis and effector mechanisms. MBio 2013;4:e000193. doi:10.1128/mBio.00193-13.

[6] Li D, Ilnytskyy Y, Kovalchuk A, Khachigian LM, Bronson RT, Wang B, et al. Crucial role for early growth response-1 in the transcriptional regulation of miR-20b in breast cancer. Oncotarget 2013;4:1373–87. doi:10.18632/oncotarget.1165.

[7] Zhou W, Shi G, Zhang Q, Wu Q, Li B, Zhang Z. MicroRNA-20b promotes cell growth of breast cancer cells partly via targeting phosphatase and tensin homologue (PTEN). Cell Biosci 2014;4:62. doi:10.1186/2045-3701-4-62.

[8] Riley KJ, Rabinowitz GS, Yario TA, Luna JM, Darnell RB, Steitz JA. EBV and human microRNAs co-target oncogenic and apoptotic viral and human genes during latency. EMBO J 2012;31:2207–21. doi:10.1038/emboj.2012.63.

[9] Dawidowska M, Jaksik R, Drobna M, Szarzyńska-Zawadzka B, Kosmalska M, Sędek Ł, et al. Comprehensive Investigation of miRNome Identifies Novel Candidate miRNA-mRNA Interactions Implicated in T-Cell Acute Lymphoblastic Leukemia. Neoplasia N Y N 2019;21:294–310. doi:10.1016/j.neo.2019.01.004.

[10] Farazi TA, Ten Hoeve JJ, Brown M, Mihailovic A, Horlings HM, van de Vijver MJ, et al. Identification of distinct miRNA target regulation between breast cancer molecular subtypes using AGO2-PAR-CLIP and patient datasets. Genome Biol 2014;15:R9. doi:10.1186/gb-2014-15-1-r9.

[11] Floyd DH, Zhang Y, Dey BK, Kefas B, Breit H, Marks K, et al. Novel Anti-Apoptotic MicroRNAs 582-5p and 363 Promote Human Glioblastoma Stem Cell Survival via Direct Inhibition of Caspase 3, Caspase 9, and Bim. PLOS ONE 2014;9:e96239. doi:10.1371/journal.pone.0096239.
