# Supplementary material for: hsa-miR-20b-5p and hsa-miR-363-3p Affect Expression of PTEN and BIM Tumor Suppressor Genes and Modulate Survival of T-ALL Cells In Vitro
Source: Cells. 2020 May 5;9(5):1137. doi: 10.3390/cells9051137 (PMC7290785; doi:10.3390/cells9051137)
Supplement: Supplementary file 1 [file cells-09-01137-s001.zip › Suplementary materials/2020-04-24 Supplementary Figures and Tables_Desciptions.docx]

hsa-miR-20b-5p and hsa-miR-363-3p affect expression of *PTEN* and *BIM* tumor suppressor genes and modulate survival of T-ALL cells *in vitro*

Monika Drobna, Bronisława Szarzyńska, Roman Jaksik, Łukasz Sędek, Anna Kuchmiy, Tom Taghon, Pieter Van Vlierberghe, Tomasz Szczepański, Michał Witt, Małgorzata Dawidowska

**SUPPLEMENTARY FIGURES AND TABLES**

**Supplementary Figure 1.** Whole total protein membranes (in Bio-Rad Stain-free technology, before incubation with primary antibody) with protein molecular weight marker (left panel) and chemiluminescent blots (right panel) for Western Blot quantification of *PTEN* protein in DND-41 cell line and BIM protein in DND-41 and CCRF-CEM cell lines.

**Supplementary Figure 2.** Representative apoptosis plots for DND-41 [A] and CCRF-CEM [B] cell lines after inhibition or mimicry of hsa-miR-20b-5p and hsa-miR-363-3p.

**Supplementary Figure 3.**

Validation of interaction between studied miRNAs and their predicted target 3′UTRs in HEK293T cell line via Dual Luciferase Reporter Assay.

WT, wild-type sequence; MUT, sequence with mutations introduced within miRNA binding site in 3′UTR; *P < .05; **P < .01; ***P < .001; ns, not significant. The graphs present the decrease of relative luciferase activity in the presence of miRNA mimic in reference to negative control. Below each graph, the predicted interaction sites in miRNAs and mRNAs are shown, with indication of nucleotides in mRNAs mutated in rescue experiment. [A] Interaction between predicted MRE in *PTEN* 3′UTR and hsa-miR-20b-5p. [B] Interaction between predicted MRE in *PTEN* 3′UTR and hsa-miR-363-3p. [C] Interaction between predicted MRE in *SOS1* 3′UTR and hsa-miR-20b-5p. [D] Interaction between predicted MRE in *BIM* 3′UTR and hsa-miR-363-3p. [E] Interaction between first predicted MRE in *FGD4* 3′UTR and hsa-miR-20b-5p. [F] Interaction between second predicted MRE in *FGD4* 3′UTR and hsa-miR-20b-5p. [G] Interaction between first predicted MRE in *FBXW7* 3′UTR and hsa-miR-363-3p. [H] Interaction between second predicted MRE in *FBXW7* 3′UTR and hsa-miR-363-3p. [I] Interaction between first predicted MRE in *NOX4* 3′UTR and hsa-miR-363-3p. [J] Interaction between second predicted MRE in *NOX4* 3′UTR and hsa-miR-20b-5p. [K] Interaction *NSMAF* 3′UTR and hsa-miR-363-3p.

**Supplementary Figure 4.** Evaluation of the expression of target genes for hsa-miR-20b-5p and hsa-miR-363-3p on mRNA and protein level after inhibition and mimicry of these miRNAs in DND-41 and CCRF-CEM T-ALL cell lines. mRNA and protein level of *SOS1* in DND-41 [A] and in CCRF-CEM [B] cell lines and of *FBXW7* in DND-41 [C] and in CCRF-CEM [D] cell lines. NC – negative control; * - p<0.05.

**Supplementary Figure 5.** Fold enrichment of hsa-miR-20b-5p, hsa-miR-363-3p, *PTEN* and *BIM* in AGO2-RIP fraction in reference to IgG-RIP (negative control) fraction, calculated as 2-ΔCq (Cq – quantification cycle), where ΔCq = Cq(AGO2-RIP) – Cq(IgG-RIP).

**Supplementary Figure 6.** Functional effect of simultaneous inhibition of hsa-miR-20b-5p and hsa-miR-363-3p as compared to negative control and to inhibition of single miRNAs on cell cycle distribution in DND-41 T-ALL cell line. * - p<0.05.

**Supplementary Table 1.** List of primers used for RT-qPCR.

**Supplementary Table 2.** List of oligonucleotides used for cloning into pmiRGLO plasmid.

**Supplementary Table 3.** miRNA-mRNA interactions for genes overrepresented in positive regulation of apoptosis GO term.

**Supplementary Table 4.** miRNA-3’UTR interactions of hsa-miR-20b-5p and hsa-miR-363-3p potentially involved in positive regulation of apoptosis.
