# Supplementary figures and images for: hsa-miR-20b-5p and hsa-miR-363-3p Affect Expression of PTEN and BIM Tumor Suppressor Genes and Modulate Survival of T-ALL Cells In Vitro
Source: Cells. 2020 May 5;9(5):1137. doi: 10.3390/cells9051137 (PMC7290785; doi:10.3390/cells9051137)

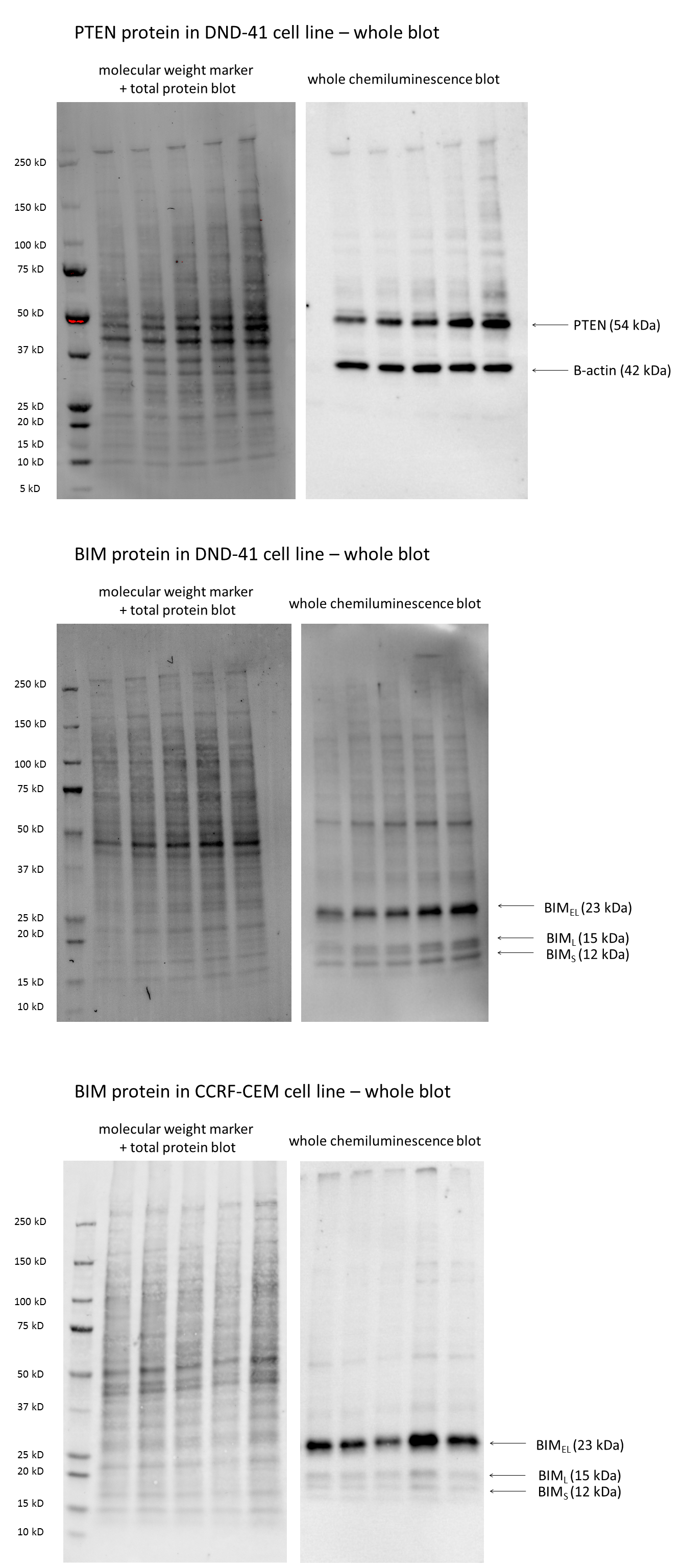

Supplement: Supplementary file 1 [file cells-09-01137-s001.zip › Suplementary materials/Supplementary Figure 1.tif]

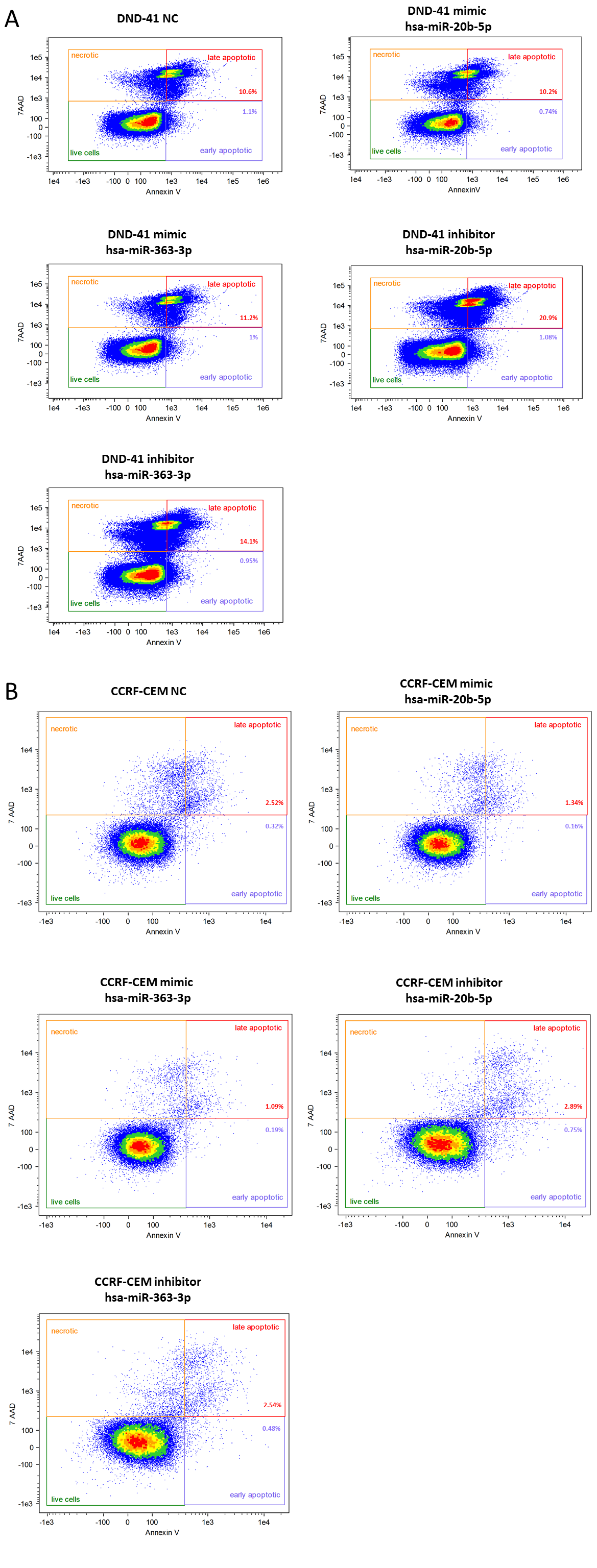

Supplement: Supplementary file 1 [file cells-09-01137-s001.zip › Suplementary materials/Supplementary Figure 2.tif]

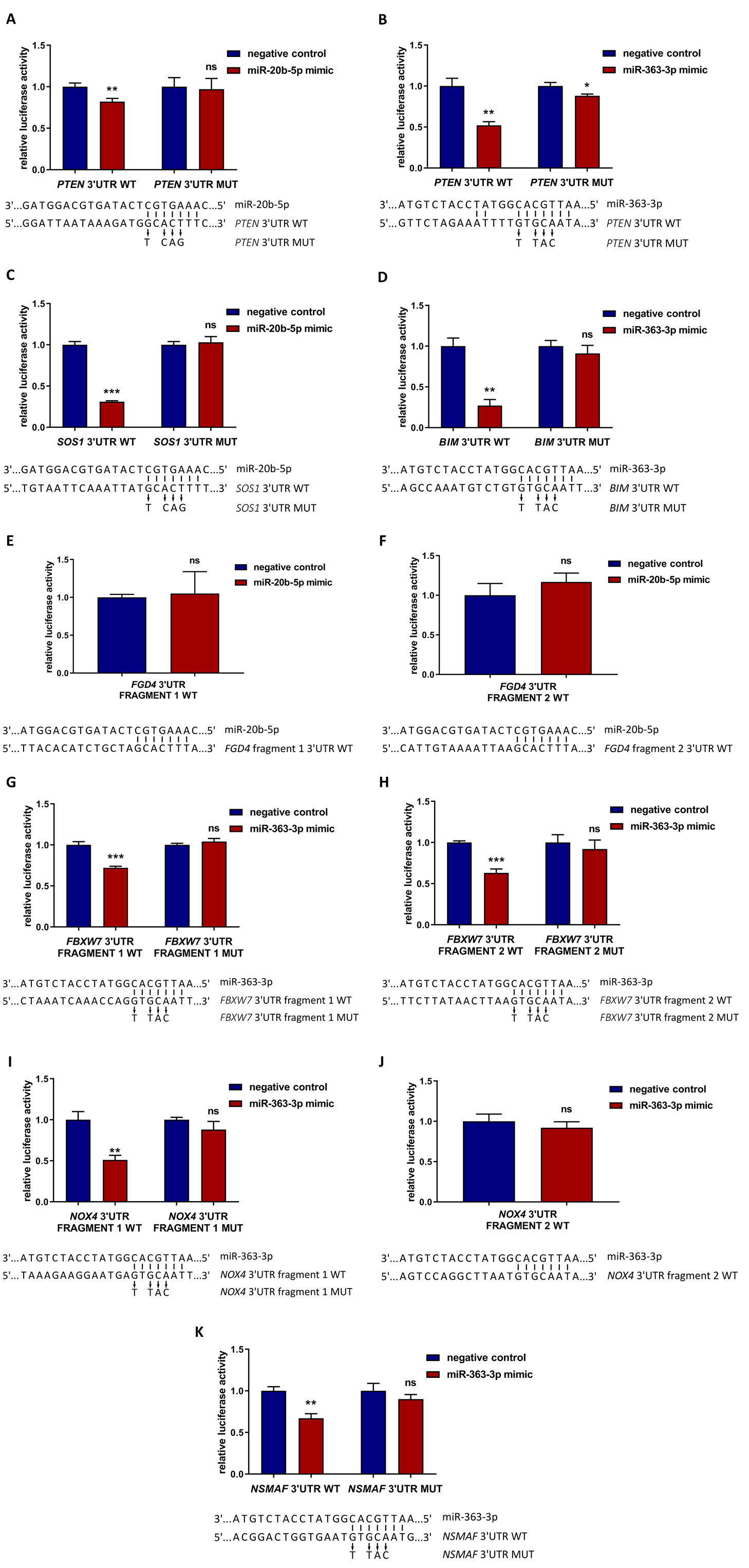

Supplement: Supplementary file 1 [file cells-09-01137-s001.zip › Suplementary materials/Supplementary Figure 3.tif]

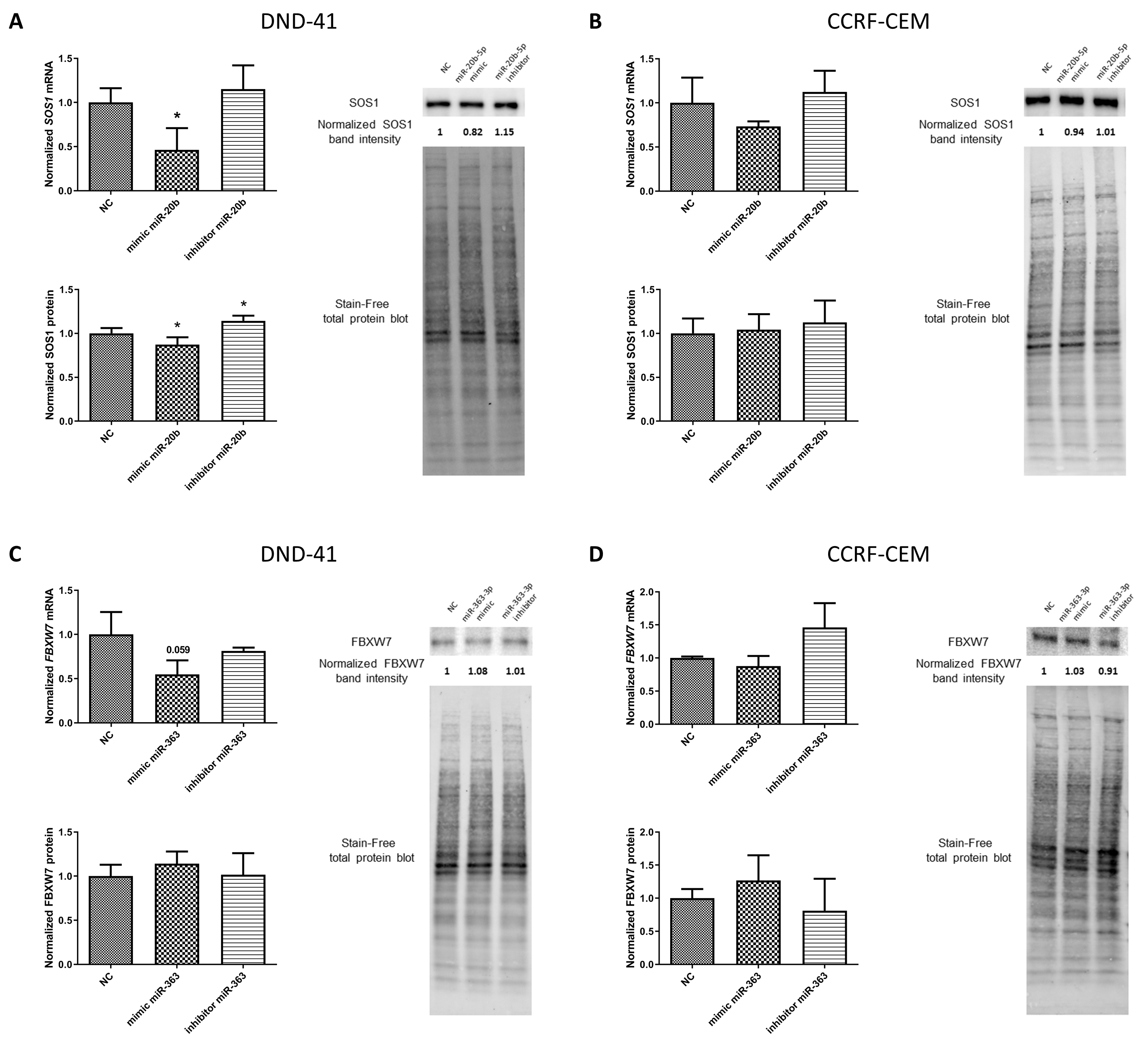

Supplement: Supplementary file 1 [file cells-09-01137-s001.zip › Suplementary materials/Supplementary Figure 4.tif]

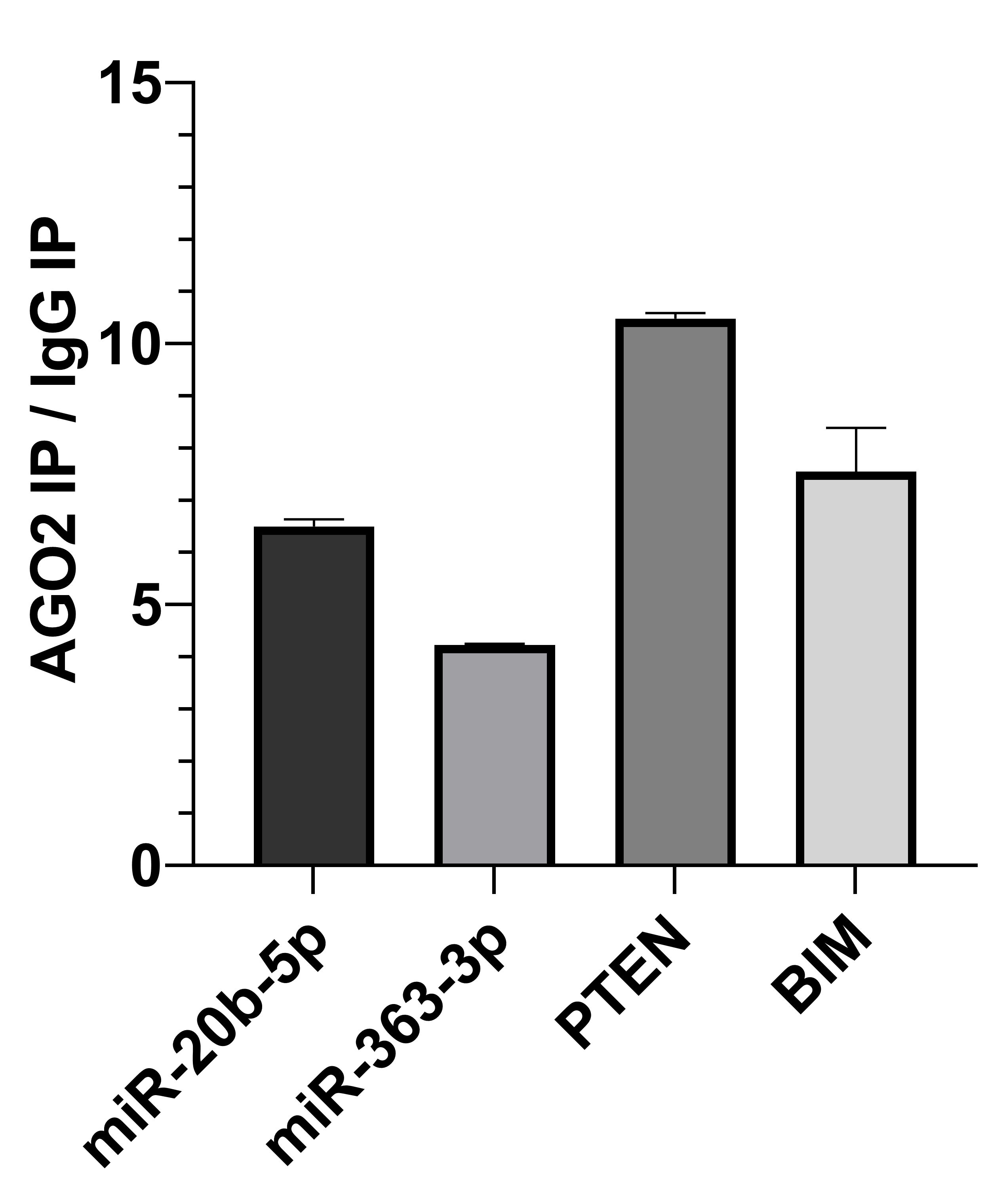

Supplement: Supplementary file 1 [file cells-09-01137-s001.zip › Suplementary materials/Supplementary Figure 5.tif]

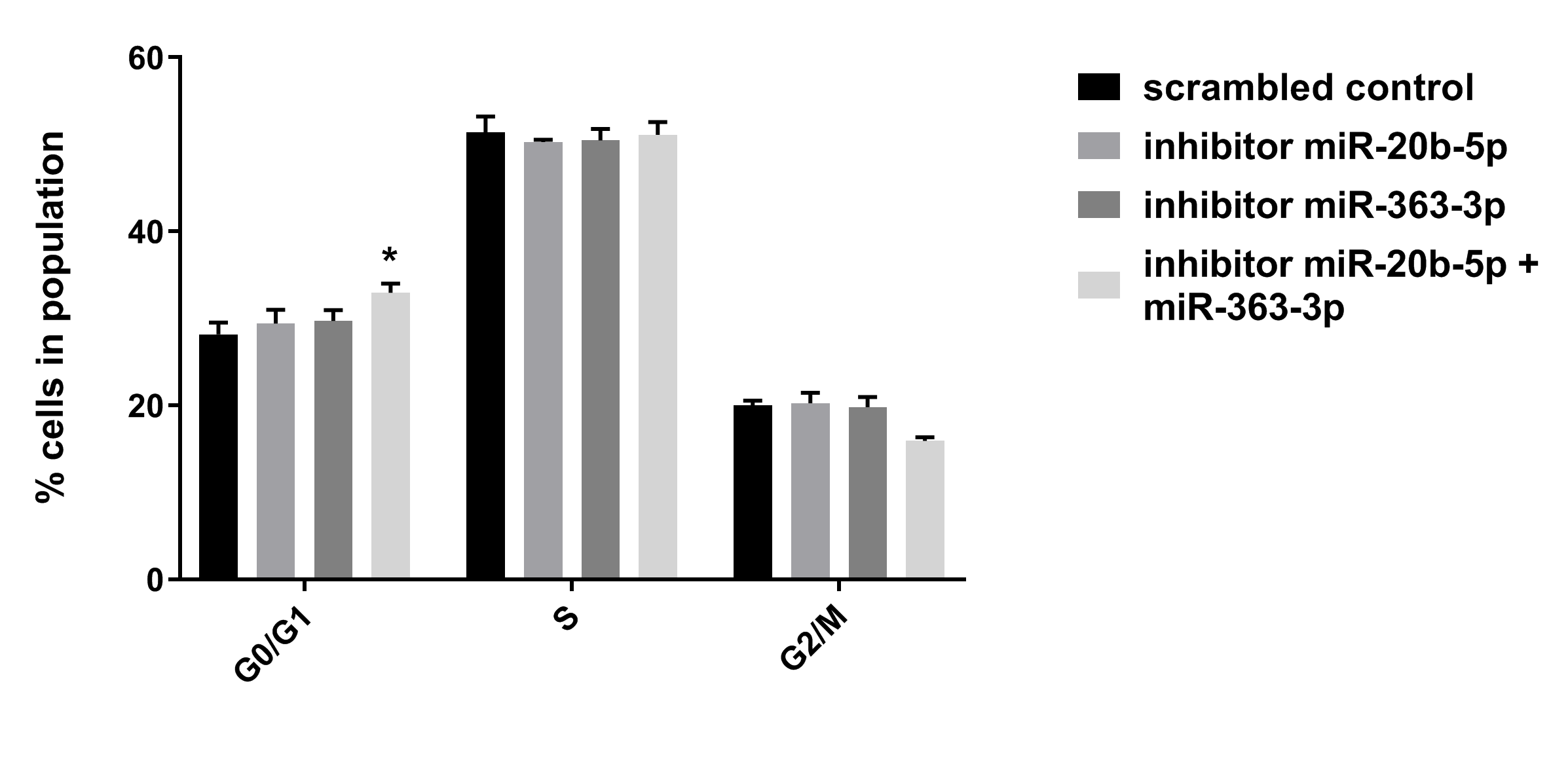

Supplement: Supplementary file 1 [file cells-09-01137-s001.zip › Suplementary materials/Supplementary Figure 6.tif]
